# Pleiotropic functions of SscA on the asexual spore of the human pathogenic fungus *Aspergillus fumigatus*

**DOI:** 10.1080/21501203.2023.2294061

**Published:** 2023-12-25

**Authors:** Ye-Eun Son, Jiwoo Han, Kyung-Tae Lee, Hee-Soo Park

**Affiliations:** a School of Food Science and Biotechnology, Kyungpook National University, Daegu, Republic of Korea; b Korea Zoonosis Research Institute, Jeonbuk National University, Iksan, Republic of Korea; c Department of Integrative Biology, Kyungpook National University, Daegu, Republic of Korea

**Keywords:** *Aspergillus fumigatus*, asexual spore, spore-specific transcription factor, C_2_H_2_ zinc finger domain, SscA, spore maturation, spore dormancy

## Abstract

Asexual spores, called conidia, are key reproductive fungal particles that enable survival in harsh environmental conditions or host systems. The conidia can infect humans, animals, and plants to cause various fungal diseases. Transcription factors, including VosA, WetA, and SscA, have key roles in conidia formation and long-term survival in *Aspergillus nidulans*. Herein, we report the pleiotropic functions of SscA in the conidia of the human pathogen *A. fumigatus*. The deletion of *sscA* increased conidia formation despite decreased fungal growth. Absence of *sscA* impaired long-term survival and reduced spore resistance to various stresses, including heat, UV, and oxidation. Transcriptomic analyses showed that SscA involved the mRNA expression of cell wall organisation-related genes. Importantly, the *sscA* deletion mutant conidia contained an increased amount of β-glucan and chitin compared to wild type conidia. In addition, conidial gliotoxin production was decreased in the *sscA* deletion strain. Overall, SscA has pleiotropic roles in conidia formation, maturation and dormancy and mycotoxin production in *A. fumigatus*.

## 1. Introduction

The *Aspergillus* species are saprotrophic filamentous fungi that decay organic materials for absorption of nutrients. These species are globally ubiquitous and are primarily spread by airborne asexual spores (conidia). *Aspergillus fumigatus* is the main opportunistic human pathogen among *Aspergillus* species. Most healthy people are unaffected by airborne *A. fumigatus* conidia, but people with impaired immunity can be threatened by *A. fumigatus* conidia, which are potent infectious particles in humans. The conidia (2–3 μm in diameter) can be inhaled into the lung thereby bypassing the immune defence mechanism; germination of conidia forms fungal hyphae to extend their niche and produce various secondary metabolites. Depending on the health of the immune system, *A. fumigatus* can cause symptoms ranging from mild fever and cough to severe and life-threatening diseases, including invasive pulmonary aspergillosis (IPA), aspergilloma, chronic pulmonary aspergillosis (CPA), allergic bronchopulmonary aspergillosis, and severe asthma with fungal sensitisation (Latge 1999; Cramer et al. 2011; van de Veerdonk et al. 2017; Earle et al. 2023). Therefore, it is necessary to understand *A. fumigatus* conidia to enable control of conidium-based *A. fumigatus* infections.

The bluish-green-coloured conidia of *A. fumigatus* start to generate from the stalks, followed by vesicles, metulae, and phialides. Asymmetric mitotic divisions of phialides arise in young conidia on conidiophores, which mature by coordinating the conidial cell wall compositions and cytoplasmic organelles (Ghiorse and Edwards 1973; Yu 2010). Separated from the conidiophores, conidia remain viable in their resting state for at least one year at room temperature until meeting favourable conditions (Lamarre et al. 2008). Dormant conidia store various transcripts required for rapid translation activity to enable spore germination (Lamarre et al. 2008; Wang et al. 2021). Conidia can also survive under hostile conditions, such as thermal, oxidative, and radiative stresses (Baltussen et al. 2019; Son et al. 2023b). Under these conditions, various regulators are involved in conidial survival. For example, *velvet* regulators VosA and VelB are essential for responding to oxidative and radiative stresses (Park et al. 2012). The mitogen-activated protein kinase SakA is required for tolerating osmotic and oxidative stress, cell wall-disturbing stress and heat shock (Bruder Nascimento et al. 2016). The bZIP transcription factor (TF) AtfA plays important roles in conidial viability under oxidative and thermal stress, dehydration and low temperatures (Hagiwara et al. 2014). The Myb-type TF MybA maintains long-term viability and controls trehalose and cell wall biosynthetic genes in conidia (Valsecchi et al. 2017). Knowledge of these genetic regulators, including TFs, kinases and phosphatases, in conidiogenesis, remains insufficient despite recent studies in the area, and further study is required to understand how fungi use conidia to proliferate and survive (Park and Yu 2016; Son et al. 2023b).

The cell wall of intact conidia is mainly composed of β-1,3-glucan, chitin, α-1,3-glucan, galactomannan and melanin, and the surface is covered with a rodlet layer (Valiante et al. 2015; Kumari et al. 2021). The most abundant polysaccharides of the cell wall are β-1,3-glucan and chitin. The β-1,3-glucan is branched and linked to chitin and galactomannan to make the conidia rigid (Fontaine et al. 2000). Several regulators are required to correctly organise β-1,3-glucan or chitin biosynthesis in the cell wall. β-1,3-glucan synthase (FksA) and β-1,3-glucanosyltransferases (Gel family) are necessary for glucan biosynthesis, and a total of nine chitin synthases are needed for chitin biogenesis in *A. fumigatus* (Fontaine et al. 2000; Brauer et al. 2023; Liu et al. 2023). The cell wall integrity signalling pathway (Bck1, Mkk, and MpkA) is involved in maintaining normal cell walls, and the downstream TFs RlmA and HsfA regulate cell wall integrity and remodelling by controlling glucan and chitin metabolism (Valiante et al. 2015; Rocha et al. 2020; Fabri et al. 2021). The conidial wall is a protective physical blockade that enables conidia to tolerate and adapt to external stressors, such as desiccation, oxidative stress, high temperatures and even hypoxic conditions in pulmonary tissue (Garcia-Rubio et al. 2019). Therefore, it is necessary to study regulators that coordinate conidial wall integrity.

Our previous studies found a gene *sscA* (spore-specific C_2_H_2_ **A**) which is commonly highly expressed in conidia of three *Aspergillus* species (*A*. *nidulans*, *A*. *flavus*, and *A*. *fumigatus*). The deletion of *sscA* resulted in defective conidia formation, impaired spore viability and increased spore stress sensitivity in *A. nidulans*. SscA also contributes to cell wall integrity and metabolism activity in *A. nidulans* conidia (Son et al. 2023a). In addition, homology of SscA in *A. fumigatus* exerted conserved roles in *A. nidulans*. However, SscA in conidia has not been studied in the human pathogenic fungus *A. fumigatus*. Herein, we characterised the roles of SscA (Afu3g09820) in conidia formation, conidial wall integrity, and gliotoxin production in *A. fumigatus*.

## 2. Materials and methods

### 2.1. Strains, media, and culture conditions

All *A. fumigatus* strains used in this study are shown in Table 1. *A. fumigatus* AF293 was used as the control strain, and *A. fumigatus* AF293.1 was used as the parental strain to construct deletion mutants. *A. fumigatus* strains were grown on minimal media with 0.1% yeast extract (MMY) as described previously (Park et al. 2012). To assay the number of conidia in wild-type (WT, AF293), *sscA*-deleted (Δ*sscA*) (TYE53.1) and complemented (Cʹ *sscA*) (TYE61.1) strains, approximately 10^6^ spores, were point-inoculated onto solid MMY and incubated at 37 °C for 4 days. For plasmid construction, *Escherichia coli* DH5α was grown in Luria-Bertani medium (BD, Difco, USA) with 100 μg/mL of ampicillin (Thermo Scientific, USA).

**Table 1.** *Aspergillus fumigatus* strains used in this study.

Strain name	Relevant genotype	References
AF293	*A. fumigatus* wild type	Brookman and Denning (2000)
AF293.1	*pyrG1*	Xue et al. (2004)
TYE53.1-3	*pyrG1*; Δ*sscA::AnipyrG*^+^	This study
TYE61.1	*pyrG1*; Δ*sscA::AnipyrG*^*+*^; *sscA(p)::sscA::FLAG*_*4x*_::*ptrA*^*R*^	This study

### 2.2. Construction of deletion mutants and complemented strains

The oligonucleotides used in this study are listed in Table 2. For generating the *sscA*-deletion mutant, the double-joint PCR method was explored (Yu et al. 2004). The fragments containing regions flanking the 5ʹ and 3ʹ ends of the *sscA* gene were amplified from *A. fumigatus* AF293 genomic DNA (gDNA) with OHS1724, OHS1725, OHS1726, and OHS1727 primers. The *A. nidulans pyrG* marker was amplified from *A. nidulans* FGSC4 gDNA with OHS2008 and OHS2009. All fragments were linked and amplified using OHS1728 and OHS1729 primers. The resulting PCR cassette was introduced into the recipient *pyrG*^−^ strain (*A. fumigatus* AF293.1) using a PEG-mediated transformation method. At least three independent deletion strains were constructed and verified by PCR and restriction enzymes (Figure S1).

**Table 2.** Oligonucleotides used in this study.

Name	Sequence (5′ → 3′)	Purpose
OHS2008	TCATCCATGGTGTCCTCGTC	*AnipyrG*_Marker_F
OHS2009	CTGGAATCAGTGGAGCGAAC	*AnipyrG*_Marker_R
OHS1724	CCTGCGACCAGATCTCTCC	5′ *sscA* DF
OHS1725	*TTTGTAGGCTTTGGGCTGTTCACAA* CTTCCGCTCTAGGAAGCGAC	3′ *sscA* with *AnipyrG* tail
OHS1726	*CTGATCTACCCCTTGGAACGCAGCA* GCAGTCCACAGCTTTCGTATG	5′ *sscA* with *AnipyrG* tail
OHS1727	AGCAACCAAGGTCAGGGT	3′ *sscA* DR
OHS1728	ATCCTGCAGTCTCGACTCC	5′ *sscA* NF
OHS1729	CCTATGACGTTGTCGACGAGA	3′ *sscA* NR
OHS1730	TCCTCACTTAGCTTCGGCAA	5′ *sscA* RT_F
OHS1731	GATGCAAAGGGATTCGCACT	3′ *sscA* RT_R
OHS1826	AATT CTGCAG GCTCCATCCGTTCCTTGTAGG	5′ *sscA* with promoter and *Pst*I
OHS1827	AATT CTGCAG CGTGGCGACTGCAACAAG	3′ *sscA* with *Pst*I
OHS2512	TCTGGGTGCTTTCTGGTTCT	5′ *fksA* RT_F
OHS2513	GCGGACCAAATCCTGTAACC	3′ *fksA* RT_R
OHS2514	CTGACTGTCCCTAGCCTGAC	5′ *gelA* RT_F
OHS2515	GACGGTGGACAGGAGTGTAA	3′ *gelA* RT_R
OHS2792	GTCGCGTTACAGAACGACAA	5′ *chsA* RT_F
OHS2793	CCAGGTACATGTTGGCTGTG	3′ *chsA* RT_R
OHS2794	GGGTACCAAGGGAGACAACA	5′ *chsB* RT_F
OHS2795	AGCCACTGTCAATGTCAAGC	3′ *chsB* RT_R
OHS2796	GGCAACTTCCTCTCCTCCTT	5′ *chsC* RT_F
OHS2797	TGCCAAGGGTCCATGTAGAG	3′ *chsC* RT_R
OHS2798	TGGATAGCGCTCGTTTACCT	5′ *chsE* RT_F
OHS2799	TCGCACATCTTTGCGCTTTA	3′ *chsE* RT_R
OHS2800	CGCCCTTCTATGAGACCAGT	5′ *gliT* RT_F
OHS2801	GCCAAAGATCCCATCGACAC	3′ *gliT* RT_R
OHS2802	GATTCTCGAGGCGTATGTGG	5′ *gliA* RT_F
OHS2803	AGACAACAAGGTCGCAATCC	3′ *gliA* RT_R
OHS2806	TCACAACCACCTATGGCAGT	5′ *rglT* RT_F
OHS2807	CTCGACGGTAATGTGAACGG	3′ *rglT* RT_R

Tail sequence is in italic.

For generating *sscA*-complemented strains, the open reading frame region of the *sscA* gene was amplified from *A. fumigatus* AF293 gDNA with primer set OHS1826 and OHS1827, digested with *Pst*I and cloned into pYES1 containing the pyrithiamine-resistant gene (*ptrA*) as a selectable marker. The cloned plasmid pYE12.1 was transformed into the recipient *sscA* strain (TYE53.1). The *sscA*-complemented transformants were selected in MMY supplemented with pyrithiamine (1 μg/mL) and confirmed by PCR and reverse-transcription-quantitative PCR (RT-qPCR) (Figure S1).

### 2.3. Phylogenetic analyses

The protein sequence of *A. fumigatus* SscA was retrieved from the FungiDB platform, and homologous proteins were identified with Basic Local Alignment Search Tool (BLASTP) searches of the National Center for Biotechnology Information (NCBI) database. A total of amino acid sequences of 80 *Aspergillus* species, including *A. fumigatus*, were aligned using Clustal W, and a phylogenetic tree was constructed with MEGA X software. The tree was generated based on the neighbour-joining method with 1,000 bootstrap replicates.

### 2.4. Transcriptome analysis

For the transcriptomic study of *A. fumigatus* WT and Δ*sscA* conidia, each strain was streaked onto solid MMY and grown at 37 °C for 2 days. Conidia of each strain were then suspended with 0.02% Triton X-100 (Thermo Scientific, USA) and filtered with Miracloth (Merck, USA).

Total RNA extraction was performed as previously described (Son and Park 2020). All samples were isolated using a Mini-bead beater (BioSpec Products Inc., USA) and TRIzol (GeneAll Biotechnology, Republic of Korea), and the extracted RNA samples were dissolved in diethylpyrocarbonate (DEPC)-treated water (Bioneer, Republic of Korea). Total RNA was treated with RQ1 RNase-free DNase (Promega, USA) and purified using the RNeasy Mini Kit (Qiagen, Germany). RNA sequencing was performed by Theragen Bio (Suwon, Republic of Korea). Briefly, three biological replicates of each strain were sequenced on Illumina Novaseq 6000, and reads were annotated with the *A. fumigatus* AF293 transcriptome using the aligner STAR v.2.3.0e software. The DESeq2 method was used for evaluating and normalising differentially expressed genes (DEGs). DEGs were screened with |log2(fold change)| ≥ 1 and *p* value < 0.05, and gene ontology (GO) analyses were performed using the R package and FungiDB database.

### 2.5. RT-qPCR analysis

RT-qPCR was performed as previously described (Son and Park 2020). The extracted RNA samples were used for the synthesis of cDNA with GoScript reverse transcriptase (Promega, USA). RT-qPCR was performed with iTaq Universal SYBR Green Supermix (BioRad, USA), synthesised cDNA and gene-specific primers on a CFX96 Touch Real-Time PCR (BioRad, USA). The expression levels of target genes were normalised to the expression levels of β-actin, an endogenous control, and calculated using the 2^−ΔΔCT^ method. Results are representative of three independent experimental replicates, and the primers used for quantitative PCR are listed in Table 2.

### 2.6. Spore viability and stress tolerance

For evaluating the long-term spore viability, 2-, 15-, and 30-day-old conidia of each strain were collected and counted using a haemocytometer. Approximately 100 conidia were spread onto solid MMY and incubated at 37 °C for 2 days. The relative survival rate was calculated as the ratio of the number of viable colonies in the 2-, 15-, and 30-day-grown conidia to the number of viable colonies in the 2-day-grown conidia.

To test spore thermal or oxidative stress tolerance, 2-day-old conidia of each strain were collected and counted using a haemocytometer. Conidia diluted by 10^3^ were heated at 55 °C for 30 or 60 min or incubated with 0.05 mol/L H_2_O_2_. Approximately 100 conidia of each strain were spread onto solid MMY and incubated at 37 °C for 2 days (Park et al. 2012).

To test spore UV stress tolerance, 2-day-old conidia of each strain were collected and counted using a haemocytometer. Conidia diluted by 10^2^ of each strain were spread onto solid MMY. Plates were irradiated directly with 50 J/m^2^ of UV light (254 nm) using a UV crosslinker (Spectrolinker XL-1000 UV crosslinker, Spectronics Corporation, USA) incubated at 37 °C for 2 days. The relative survival rate was calculated as the ratio of the number of viable colonies in the treated sample to the number of viable colonies in the untreated control. Spore viability and stress tolerance assays were replicated at least three times.

### 2.7. Spore β-glucan and chitin analysis

β-glucan in spores was assayed using a Glucatell ® assay kit (Associates of Cape Cod Inc., USA) as described previously (Park et al. 2015). The 2-day-old conidia of each strain were collected, diluted by 10^3^, and resuspended in 25 μL. Each sample was mixed with Glucatell ® reagent and incubated at 37 °C for 30 min. The reaction was stopped using diazo coupling reagents, and the optical density of each sample was measured at 540 nm. The spore β-glucan assay was replicated at least three times.

Chitin was extracted from conidia and analysed as previously described (Lehmann and White 1975) with some modifications. The 2-day-old (2 × 10^9^) conidia of each strain were collected, resuspended with 3% sodium dodecyl sulphate (Bioneer, Republic of Korea) and heated at 100 °C for 15 min. After being washed with ddH_2_O, saturated potassium hydroxide (Thermo Scientific, USA) was added and heated at 100 °C for 1 h. Then, 75% ice-cold ethanol (Merck) and 5% Celite 545 suspension (Samchun, Republic of Korea) were added and centrifuged at 4 °C. Each sample was washed with 40% ice-cold ethanol and ddH_2_O and adjusted to pH 2. After centrifuging at 4 °C, the supernatant was decanted and the pellet was suspended in the same volume of water: 5% sodium nitrite (Sigma, USA): 5% potassium hydrogen sulphate (Junsei Chemical, Japan). Samples were incubated at room temperature for 15 min and centrifuged at 4 °C, and 20 μL of 12.5% ammonium sulfamate (Sigma, USA) was added, and the mixture was incubated at room temperature for 5 min. After that, 20 μL of 0.5% 3-methylbenzthiazolinone-2-hydrazone (Thermo Scientific, USA) were added and incubated at 100 °C for 3 min. Finally, 20 μL of 0.83% ferric chloride (Sigma, USA) was added and incubated at room temperature for 30 min, and the optical density of each sample was measured at 650 nm. The negative control was distilled water, and the positive control was 1% glucosamine (Thermo Scientific, USA). The chitin assay was replicated at least three times.

### 2.8. Spot assay for cell wall stresses

For a spot dilution assay, 2-day-old conidia of each strain were collected and counted using a haemocytometer. Conidia suspensions of each strain, starting with 2 × 10^6^ conidia/mL, were serially ten-fold diluted and spotted on solid MMY containing cell wall-stressing reagents: 10, 50, or 100 μg/mL of Congo red (CR) and calcofluor white (CFW). All plates were incubated at 37 °C for 48 h and then photographed (Schruefer et al. 2021).

### 2.9. Gliotoxin (GT) analysis

GT production was analysed by thin-layer chromatography (TLC) as described previously with minor modifications (Xiao et al. 2010). The 7-day-grown conidia (1 × 10^10^) of each strain were collected and homogenised using a Mini-Bead beater (BioSpec Products Inc.) with 0.3 mL of glass beads (Daihan Scientific, Republic of Korea) and 1 mL of chloroform (Sigma, USA). The organic phase was mixed with ddH_2_O and centrifuged. The purified organic phase was transferred to a new glass vial and dried at 65 °C overnight. Samples were resuspended with 0.1 mL of methanol (Honeywell, USA), spotted onto a TLC silica plate coated with the fluorescent indicator F254 (Merck Millipore, USA), and resolved in TLC plates containing toluene (Daejung, Republic of Korea): ethyl acetate (Daejung, Republic of Korea): acetic acid (Thermo Fisher, USA) (5:4:1, v/v/v). The TLC plate was exposed and captured under UV light at 254 nm. The relative intensities of GT were calculated using the ImageJ software.

### 2.10. *In vivo A. fumigatus* infection

Six-week-old Balb/c female mice were used for the experiment after a one-week stabilisation period (*n* = 7). For immunosuppression, 250 mpk of cyclophosphamide was orally administered before infection (3 days, 1 day before), and 125 mpk of cyclophosphamide was orally administered after infection (1, 6, and 12 days after). One day before infection, 250 mpk of cortisone acetate was also orally administered. 1 × 10^7^ conidia of each strain were anaesthetized with isoflurane and then nasally inhaled. After infection, the condition of the mice was observed every day, and the survival rate of the mice was expressed as percent survival. All experiments followed the experimental ethics guidelines by the Institutional Animal Care and Use Committee of the Experimental Animal Center at Jeonbuk National University (Approval number JBNU 2023-132). The survival curves were analysed using the Log-rank (Mantel-Cox).

### 2.11. Statistical analysis

Statistical differences between WT and Δ*sscA* strains were evaluated with Student’s unpaired t-test. Data are reported as mean ± standard deviation. *p* values < 0.05 were significant.

## 3. Results

### 3.1. *SscA in* Aspergillus *species*

**Figure 1.**
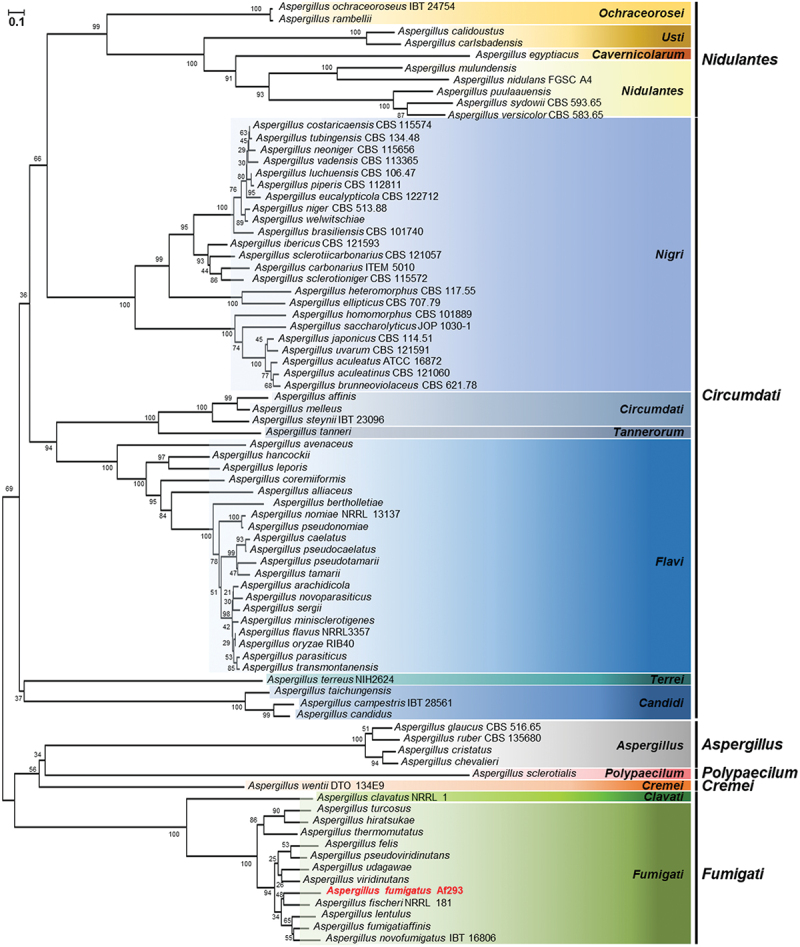
*In silico* analysis of SscA in *Aspergillus* species. a phylogenetic tree of SscA homologues identified in *Aspergillus* species. Protein sequences were obtained from the NCBI database, and alignment was performed with clustal W. The tree was generated via MEGA X to examine SscA homologues. The tree based on the neighbour-joining method was replicated 1,000 times, and the scale represented the number of substitutions per site.

The open reading frame of *sscA* (*Afu3g09820*), comprising 2,100 nucleotides with two exons and an intron, was predicted to encode a protein with 679 amino acids and an estimated mass of 73.2 kDa. SscA has been indicated to be highly conserved in diverse phylum of fungi (Son et al. 2023a). To understand the role of SscA in *Aspergillus* species, we identified the homologues of SscA among 80 *Aspergillus* species via the BLASTP tool in NCBI and analysed the phylogenetic relationship (Table S1). SscA homologues were conserved in most *Aspergillus* species and subdivided along the subgenus and section (Figure 1). All 80 homologues of SscA were predicted to contain a Cys_2_His_2_ zinc finger domain with the C-X_(5)_-C-X_(12)_-H-X_(3)_-H present in the N-terminus in all proteins, although some amino acids differed according to section (Figure S2).

### 3.2. SscA affects vegetative growth and conidia formation

**Figure 2.**
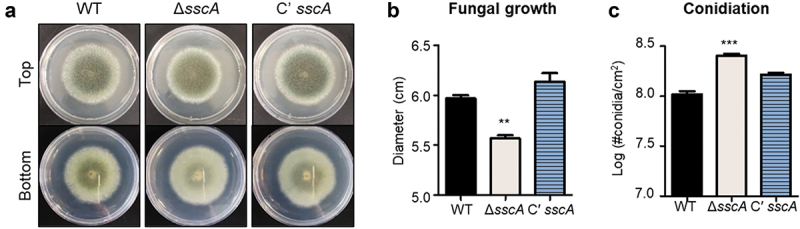
Functions of SscA in *Aspergillus fumigatus* development. (a) Photographs of colonies of WT, Δ*sscA* and Cʹ *sscA* strains point-inoculated onto solid MMY and grown at 37 °C for 4 days. (b) Quantitative analysis of fungal growth of strains shown in (a); error bars indicate the standard error of the mean in three biological replicates (**p < 0.01). (c) Quantitative analysis of asexual spore formation of the strains shown in (a); error bars indicate the standard error of the mean in three biological replicates (**p < 0.01).

To investigate the functions of SscA in *A. fumigatus*, Δ*sscA* and Cʹ *sscA* strains were point-inoculated onto solid MMY and incubated at 37 °C for 4 days. As shown in Figure 2a, the deletion of *sscA* resulted in the formation of light-coloured colony. While the fungal colony of the Δ*sscA* mutant was slightly decreased in size compared with that of the WT and Cʹ *sscA* strains, the number of conidia per cm^2^ in Δ*sscA* strain was increased compared with that in the WT and Cʹ *sscA* strains (Figure 2b–c). However, the germination rate of Δ*sscA* conidia was not different compared with other strains (Figure S3). These results suggested that SscA is required for normal fungal growth and conidiation in *A. fumigatus*.

### 3.3. SscA is involved in long-term viability and stress tolerance

**Figure 3.**
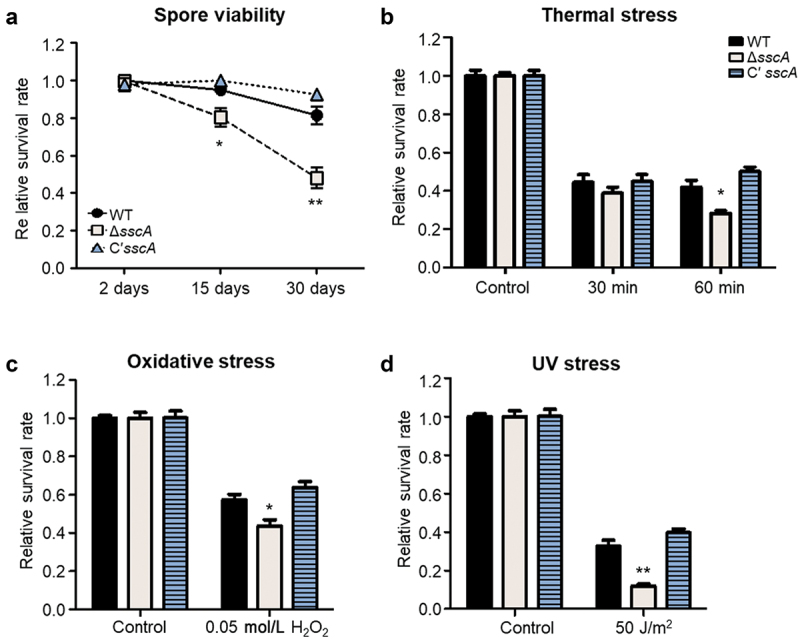
Roles of SscA in *Aspergillus fumigatus* conidia. (a) The relative survival rates of WT, Δ*sscA* and Cʹ *sscA* conidia grown for 2-, 15-, or 30-days (**p < 0.01, *p < 0.05). (b–d) The relative conidial survival rates of the designated strains treated with thermal stress (55 °C) for 30 or 60 min (b), oxidative stress (0.05 mol/L H_2_O_2_) for 30 min (c), or UV stress (50 J/m^2^) (d); error bars indicate the standard error of the mean in three biological replicates (**p < 0.01, *p < 0.05).

Our previous study demonstrated that SscA is required for conidial dormancy and stress resistance in the model organism *A. nidulans* (Son et al. 2023a). To further explore whether the functions of SscA are conserved in the human pathogen *A. fumigatus* conidia, we assayed the viability with 2-, 15-, and 30-old conidia of each strain. Although the survival rates of the WT and Cʹ *sscA* strains did not fluctuate at 15 and 30 days, the survival rates of Δ*sscA* mutant showed a significant decrease over time (Figure 3a). Next, we examined the conidial survival rates under various stresses. The survival rate of Δ*sscA* conidia was similar to that of WT and Cʹ *sscA* conidia when heated at 55 °C for 30 min (Figure 3b). However, the viability of Δ*sscA* conidia was significantly reduced compared with that of the WT and Cʹ *sscA* strains when heated for 60 min (Figure 3b). In addition, Δ*sscA* conidia were more sensitive than either WT or Cʹ *sscA* conidia when treated with oxidative and UV stresses (Figure 3c–e). Thus, SscA plays an important role in spore viability and stress tolerance in *A. fumigatus* conidia.

### 3.4. Genome-wide transcriptome analysis of SscA in *A. fumigatus* conidia

**Figure 4.**
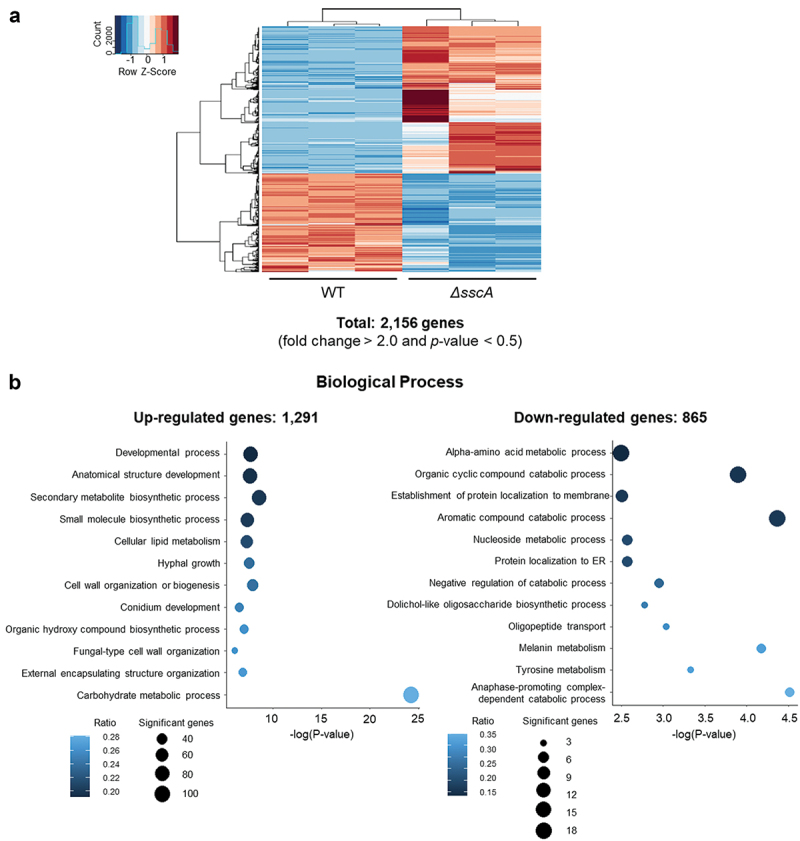
Transcriptomic analyses of Δ*sscA* conidia. (a) Heatmap showing DEGs between WT and Δ*sscA* conidia. (b) GO analyses of upregulated DEGs (left; 1,291 genes) and downregulated (right; 865 genes) DEGs.

To gain further insight into the functions of SscA in *A. fumigatus* conidia, we conducted RNA sequencing with WT and Δ*sscA* conidia grown for 2 days. Among the predicted 10,130 *A. fumigatus* genes, a total of 2,156 (|fold change| ≥ 2.0; *p* value < 0.05) were differentially expressed between WT and Δ*sscA* conidia (Figure 4a). The expression of approximately 12.7% and 8.5% of the genes were up- and down-regulated in Δ*sscA* conidia, respectively. We then performed GO term enrichment analyses based on the FungiDB platform (Figure 4b, Table S2). The up-regulated genes were associated with the developmental process, anatomical structure development, secondary metabolite biosynthetic process, cell wall organisation or biogenesis, external encapsulating structure organisation, hyphal growth, and conidium development, whereas the down-regulated genes were involved in the alpha amino acid metabolic process, organic cyclic compound catabolic process, nucleoside metabolic process, protein localisation to membrane, and the endoplasmic reticulum. These suggested that SscA influences mRNA expression of various genes associated with development, cellular metabolism, and cell wall composition in *A. fumigatus* conidia.

### 3.5. SscA is required for beta-glucan and chitin biosynthesis

**Figure 5.**
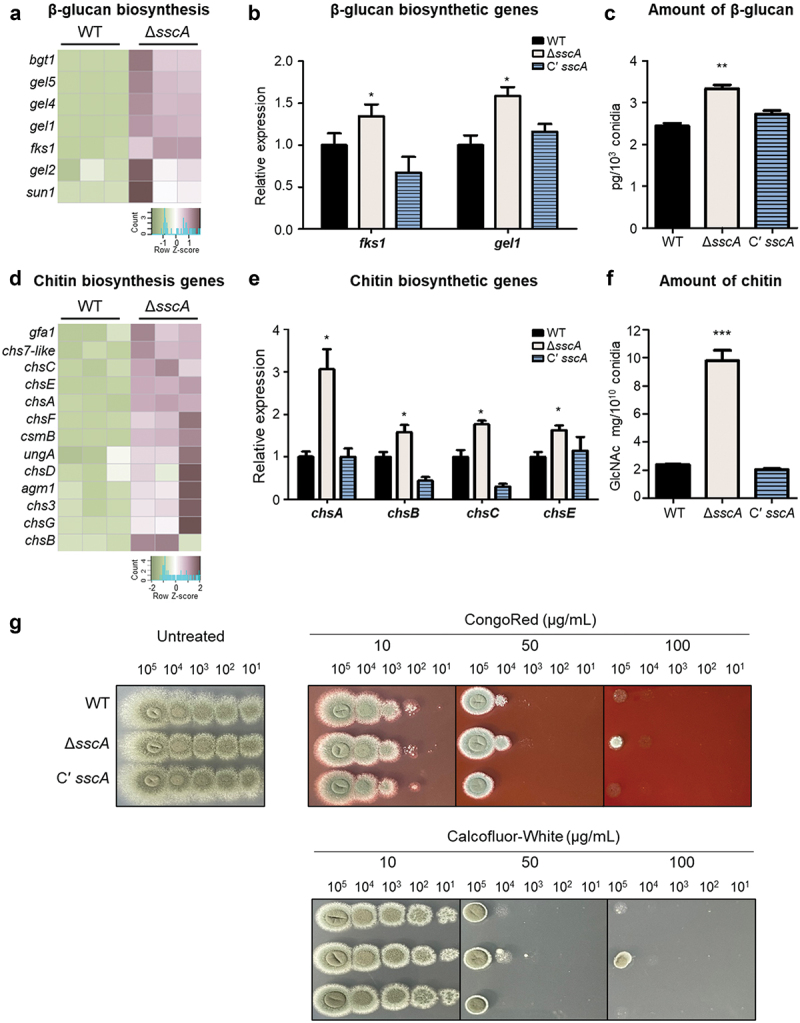
Effect of SscA on key conidial wall components in *Aspergillus fumigatus*. (a) Heatmap showing the DEGs among β-glucan biosynthetic genes in WT and Δ*sscA* conidia. (b) The relative mRNA expression levels of major β-glucan biosynthetic genes (*fksA* and *gelA*) in WT, Δ*sscA*, and Cʹ *sscA* conidia as assessed by RT-qPCR (*p < 0.05). (c) Amount of β-glucan in conidia of WT, Δ*sscA,* and Cʹ *sscA* strains (**p < 0.01). (d) Heatmap showing the DEGs among chitin biosynthetic genes in WT and Δ*sscA* conidia. (e) The relative mRNA expression levels of four chitin biosynthetic genes (*chsA*, *chsB*, *chsC*, and *chsE*) in WT, Δ*sscA*, and Cʹ *sscA* conidia as assessed by RT-PCR (*p < 0.05). (f) Amount of chitin in conidia of WT, Δ*sscA*, and Cʹ *sscA* strains (***p < 0.001). (g) Sensitivity of the designated strains to cell wall disturbing agents. The error bars indicate the standard error of the mean in three biological replicates.

SscA is indispensable for normal cell wall structure in *A. nidulans* conidia (Son et al. 2023a), and our transcriptomic data indicated that deletion of *sscA* affected gene expression of cell wall biogenesis in *A. fumigatus* conidia, suggesting that SscA can affect β-glucan and chitin biosynthesis. First, we further checked RNA-seq results and found that the mRNA expression levels of β-glucan biosynthetic genes in WT and Δ*sscA* conidia (Baltussen et al. 2019; Garcia-Rubio et al. 2019) were more highly expressed in Δ*sscA* conidia than in those of the WT conidia (Figure 5a). We then verified the RNA-seq results through the RT-qPCR analysis and confirmed that the transcripts of *fksA* and *gelA*, the key β-glucan biosynthetic genes, were upregulated in the Δ*sscA* conidia (Figure 5b). Furthermore, the β-glucan level in Δ*sscA* conidia was significantly increased compared with those of the WT and Cʹ *sscA* conidia (Figure 5c).

We then analysed the mRNA levels of chitin biosynthetic genes and found that the expression of most of the chitin biosynthetic genes was upregulated in the Δ*sscA* conidia (Figure 5d). The RT-qPCR analysis verified that the relative expression levels of four *chs* genes were significantly increased in the Δ*sscA* conidia compared with that in the WT and Cʹ *sscA* conidia (Figure 5e). We also determined that the amount of hydrolysed N-acetylglucosamine, a monomer unit of chitin, in the Δ*sscA* conidia was approximately 4-fold that in other strains (Figure 5f), suggesting that SscA is required for proper chitin biosynthesis in conidia.

We then investigated whether SscA influences sensitivity to well-known cell wall-stressing agents such as CR and CFW that mainly bind to β-1,3-glucan and chitin, respectively (Ram and Klis 2006). Sensitivity to CR and CFW was determined by a spot assay. The serially diluted, 2-day-grown conidia were spotted onto MMY medium with CR or CFW and incubated at 37 °C for 2 days. The sensitivity of the Δ*sscA* strains was similar to that of WT and Cʹ *sscA* strains at a low concentration of CR (10 μg/mL) or CFW (10 μg/mL) (Figure 5g). However, the Δ*sscA* strain showed a slightly elevated resistance to 50 μg/mL of CR and CFW, and only the Δ*sscA* strain developed colonies under high concentrations of CR (100 μg/mL) or CFW (100 μg/mL). The mRNA expression levels of α-glucan biosynthetic genes, another fundamental component of the fungal cell wall, were also upregulated in Δ*sscA* conidia (Figure S4). Overall, these results suggest that SscA is critical for conidial wall composition by coordinating expression of cell wall organisation-related genes in *A. fumigatus*.

### 3.6. SscA contributes to GT production

**Figure 6.**
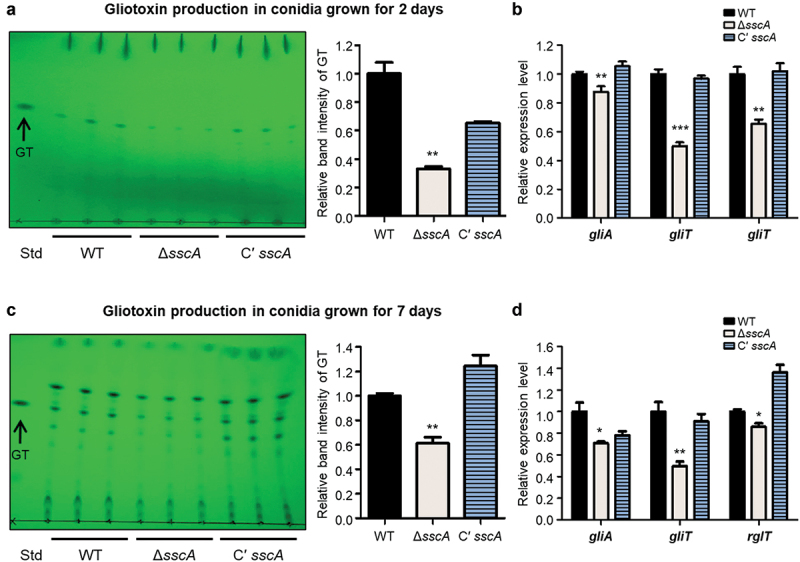
Functions of SscA in gliotoxin (GT) production. (a) TLC of gliotoxin from the 2-day-grown conidia of WT, Δ*sscA*, and Cʹ *sscA* strains. The right bar plot indicates the relative band intensity of gliotoxin shown in TLC plate; error bars indicate the standard error of the mean in three biological replicates (**p < 0.01). (b) The relative mRNA expression levels of gliotoxin biosynthetic genes (*gliA*, *gliT*, and *rglT*) in WT, Δ*sscA* and Cʹ *sscA* conidia grown for 2 days (***p < 0.001, **p < 0.01). (c) TLC of gliotoxin from the 7-day-grown conidia of WT, Δ*sscA*, and Cʹ *sscA* strains. The right bar plot indicates the relative band intensity of gliotoxin shown in TLC plate; error bars indicate the standard error of the mean in three biological replicates (**p < 0.01). (d) The relative mRNA expression levels of gliotoxin biosynthetic genes (*gliA, gliT*, and *rglT*) in WT, Δ*sscA*, and Cʹ *sscA* conidia grown for 7 days (**p < 0.01, *p < 0.05).

GT is a sulphur-containing secondary metabolite that is the major and most potent mycotoxin produced by *A. fumigatus* (de Castro et al. 2022). The biosynthesis and secretion of GT are regulated by the GT biosynthetic gene cluster including the final oxidoreductase GliT, efflux pump GliA, and TF RglT (Schrettl et al. 2010; Wang et al. 2014; Ries et al. 2020). RNA-seq data found that deletion of *sscA* affected mRNA expression of genes involved in secondary metabolism (Figure 4b and Table S3). To investigate the functions of SscA in GT production, we collected 2- or 7-day old conidia (10^10^) of each strain and extracted the fungal metabolites. The total organic metabolites were separated from fungal metabolites in a thin-layer silica gel. The Δ*sscA* conidia produced slightly less GT than either WT or Cʹ *sscA* conidia (Figure 6a). The relative band intensity of GT in Δ*sscA* was significantly decreased compared with that of WT or Cʹ *sscA* strains. Furthermore, we confirmed that mRNA expression of GT-related genes (*gliA*, *gliT*, and *rglT*) was downregulated in Δ*sscA* compared with that in WT and Cʹ *sscA* (Figure 6b). The 7-day-old conidia showed similar results, with the Δ*sscA* strain exhibiting decreased production of GT and mRNA expression of GT synthetic-related genes (Figure 6c–d). To further examine the function of SscA in GT biosynthesis in mycelia, WT and mutant strains were cultured in liquid MMY media and checked GT production. The Δ*sscA* exhibited a considerably reduced abundance of GT at 7 days compared with that of the other strains (Figure S5). Overall, these suggested that SscA affects GT production in *A. fumigatus*.

### 3.7. SscA influences the pathogenicity of *A. fumigatus*

**Figure 7.**
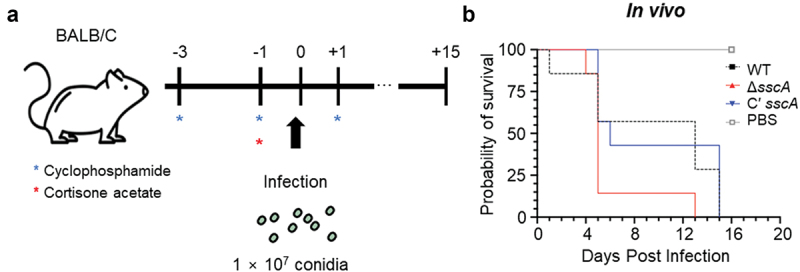
Functions of SscA in the virulence of *Aspergillus fumigatus*. (a) Schematic representation of fungal infection in the mice model. (b) Survival rate of *A. fumigatus* WT, Δ*sscA*, and Cʹ *sscA* strains in mice model. Statistical differences were calculated using the Log-rank (mantel-cox) test (WT *vs.* KO, 0.1328; WT *vs.* complemented, 0.6767; KO *vs.* complemented, 0.0483).

*A*. *fumigatus* is the primary human pathogenic fungus that causes symptoms ranging from cough and fever to severe aspergillosis, depending on the immune status of the host (Dagenais and Keller 2009). In other to investigate the functions of SscA on the virulence of *A. fumigatus*, we collected 2-day old conidia (10^7^) of each strain and intranasally infected them to mice immunosuppressed by cyclophosphamide and cyclophosphamide (Figure 7a). As a result, the mortality of mice infected with Δ*sscA* conidia was rather higher than that of mice infected with WT strain, but this was not statistically significant (Figure 7b). Therefore, this result proposes that SscA does not seem to have a significant effect on fungal pathogenicity in hosts.

## 4. Discussion

*A*. *fumigatus* is a primary causative agent of fungal-related diseases. Host species are continually exposed as *A. fumigatus* is inhaled through the respiratory tract and can infect the lungs, causing various diseases and even death (Lin et al. 2001; Brown et al. 2012). Conidia are the main infectious particles that are easily dispersed through the air, and therefore it is worthwhile seeking to control conidia viability and resistance (Nayak et al. 2018). We previously demonstrated that the spore-specific C_2_H_2_ zinc finger SscA TF is essential for conidial formation, maturation and dormancy in *A. nidulans* (Son et al. 2023a). In the current study, we investigated the roles of SscA in *A. fumigatus* conidia. The absence of *sscA* induced abnormal conidia formation, reduced long-term spore viability, and increased conidial stress sensitivity in *A. fumigatus*. Transcriptome studies indicated that SscA plays a key role in maintaining proper conidial wall components by regulating the expression of cell wall biosynthetic genes. In addition, SscA affected GT production in *A. fumigatus* conidia.

Previous studies have revealed that the defective phenotypes of Δ*AnisscA* in asexual development and conidial physiology are restored when *Afu*SscA is expressed in *A. nidulans* (Son et al. 2023a). Herein, we investigated whether such multiple roles of SscA in conidiogenesis are indeed conserved in *A. fumigatus*. First, as in *A. nidulans*, Δ*sscA* conidia showed defective long-term viability compared with WT and Cʹ *sscA* conidia in *A. fumigatus*. Deletion of *sscA* increased the sensitivity of conidia to thermal, oxidative and UV stresses (Figure 3). Although the *A. nidulans* Δ*sscA* strain did not affect fungal growth and showed defective conidia formation, the *A. fumigatus* Δ*sscA* strain exhibited reduced fungal growth and increased conidia formation (Figure 2). Collectively, the functions of SscA on conidial viability and stress response are conserved but have different roles in fungal growth and conidia formation in different *Aspergillus* species.

There are many virulence factors, including conidial tolerance, conidial surface and cell wall, pigment, and secondary metabolites in *A. fumigatus* (Hohl and Feldmesser 2007; Raffa et al. 2019). Our RNA-sequencing analysis showed that a variety of genes associated with the secondary metabolite biosynthetic process was upregulated in Δ*sscA* conidia (Figure 4b). To predict the effect of SscA on secondary metabolism, we further investigated all DEGs in 33 secondary metabolite gene clusters of *A. fumigatus* (Table S3). There were five upregulated secondary metabolites where the ratio of DEGs per secondary metabolite gene cluster was >60% (Figure S6): cluster 2 (nidulanin-like), cluster 3 (ferricrocin), cluster 15, cluster 18 (trypacidin), and cluster 26 (fumiquinazoline C). Although the bioactivities of the other metabolites have not been investigated yet, trypacidin is known as a conidia-bound metabolite that lowers the recognition or uptake of conidia by macrophages (Mattern et al. 2015). Fumiquinazoline, which accumulated in *A. fumigatus* conidia, is also a cytotoxic peptidyl alkaloid with antibacterial and antifungal activities. Taken together, we speculated that Δ*sscA* conidia may cause hypervirulence in humans, animals, and microorganisms by regulating gene expression of genes involved in specific secondary metabolites.

*A*. *fumigatus* is a saprophytic fungus that adapts and survives under various stressful conditions, including in different environments and hosts. To sustain their viability, *A. fumigatus* uses several strategies that involve trehalose, heat-shock proteins, and cell wall integrity (Al-Bader et al. 2010; Tiwari et al. 2015; Brown and Goldman 2016; Baltussen et al. 2019). To understand the regulatory roles of SscA in stress tolerance, we examined the amount of trehalose in the conidia of each strain. The amount of trehalose was slightly increased in Δ*sscA* conidia compared with that in conidia of WT and Cʹ *sscA* strains (Data not shown). To gain insights into the stress sensitivity of Δ*sscA* conidia, we also analysed the expression levels of heat-shock proteins (HSPs) based on transcriptome data. Among the various sizes of HSPs, the expression of relatively small HSPs (sHSPs) was downregulated in Δ*sscA* conidia (Figure S7A). Several studies have indicated that sHSPs play major roles in various stress responses in fungi (Tiwari et al. 2015; Wu et al. 2016; Dev 2021). Furthermore, the expression of DNA repair-related genes was downregulated in Δ*sscA* conidia (Figure S7B). Altogether, SscA controls resistance to various stresses by regulating the expression of stress-related genes in *A. fumigatus* conidia.

Fungal cell walls with both flexible and rigid properties can be remodelled depending on the internal and external environment and play as protective barriers that withstand extremely unfavourable conditions (Latge 2007; Gow et al. 2017). However, it is difficult to demonstrate a direct relationship between the cell wall organisation and sensitivity to various stresses. Deletion of *sscA* resulted in increased β-glucans and chitin contents and increased resistance in cell wall perturbing agents (Figure 5). However, Δ*sscA* showed increased sensitivity in thermal, oxidative, and radiative stresses (Figure 3b–d). Similar to SscA, deletion of *nsdC* exhibited increased cell wall thickness and mannose and galactose, but increased sensitivity to cell wall stressors (Alves de Castro et al. 2021). The Δ*rlmA* also showed increased cell wall thickness, and β-1,3-glucan and chitin levels, but it was more sensitive to cell wall damaging compounds (CR, CFW, and caspofungin), cell membrane disturbing agent (SDS), chelating agent (EDTA), and oxidative stressors (paraquat, menadione, and t-butyl hydroperoxide) (Rocha et al. 2016). The Δ*nikA* exerted a thinner cell wall and reduced galactosamine contents compared to WT and C’ *nikA*, but deletion of *nikA* strain was more resistance to CR, CFW and micafungin and less resistance to high osmotic stresses including NaCl and KCl (Hagiwara et al. 2013). Overall, these imply that the alteration of conidial wall components influences the different responses under varying stresses, suggesting adaptation to the extracellular environment in *A. fumigatus* involves not only cell wall composition but also other robust and complex signalling pathways.

Importantly, SscA is a highly conserved TF in *Aspergillus* species (Figure 1) as well as in other fungi (Son et al. 2023a), and its homologous proteins influence fungal pathogenicity. Usv101 in *Cryptococcus neoformans* is a key regulator for full pathogenesis that regulates melanin production and capsule growth. A *usv101*Δ strain exhibited increased capsule thickness and size, thereby preventing phagocytotic engulfing and delaying mouse infection and death (Gish et al. 2016). In *Candida albicans*, Bcr1 is required for the fungal burden by regulating biofilm formation in mice models (Dwivedi et al. 2011; Fanning et al. 2012). Nsf1 in *Fusarium graminearum* and Gcf6 in *Magnaporthe oryzae* play vital roles in full virulence in wheat and rice, respectively (Cao et al. 2016; Shi et al. 2021). Similar to other fungal species, SscA has an effect on the pathogenicity of *A. fumigatus* in mice models (Figure 7). Previous studies demonstrated that *ΔdvrA* (*ΔsscA*) had increased virulence in the nonneutropenic mice model as well as the *Galleria melonella* model and suggested that the hypervirulence in mice infected with *ΔdvrA* was induced by augmented pulmonary fungal burdens (Ejzykowicz et al. 2010). However, DvrA did not affect pathogenicity in the neutropenic mice model, which differed from our results. We speculate on two possibilities for these distinct results. First, we intranasally injected fungal spores directly, whereas the Ejzykowicz group infected mice in fungal spores-aerosolized chambers for 1 h; thus, because of these different experimental methods, the actual concentration of infected fungal spores may have varied. Second, differences were present in the amount and frequency of immunosuppressive drugs used. Nevertheless, with the previous and current studies, we suggest that *Afu*SscA, unlike other SscA homologues in other fungi, is important for the regulation of pathogenicity in hosts.

Notably, *A. fumigatus* produces multiple virulence factors, including an external encapsulating structure and fungal mycotoxins and exhibits thermotolerance, antioxidant defence mechanisms and adaptation to hypoxic conditions (Tomee and Kauffman 2000; Hohl and Feldmesser 2007; Abad et al. 2010). The fungal cell wall is closely related to the external environment and can evoke the host immune system when recognised as pathogen-associated molecular patterns. For example, β-glucan is recognised by the C-type lectin receptor dectin-1 and induces an inflammatory response (Hohl et al. 2005; Hopke et al. 2018). Chitin stimulates inflammatory immune reactions through recognition via the toll-like receptor 2 and dectin-1 (van de Veerdonk et al. 2017; Elieh Ali Komi et al. 2018). The infected conidia can be engulfed by phagocytes (mainly alveolar macrophages), which subsequently induce inflammatory reactions and excessive immune responses ranging from asthma to CPA and IPA. Herein, we determined that SscA is critical for regulating correct conidial wall dynamics in *A. fumigatus*. As shown in Figure 5, the amounts of β-glucan and chitin, the main cell wall constitutions, were significantly increased in the conidia of Δ*sscA* strain. Previous studies suggested that large-sized cells of *C. neoformans* and *C. albicans* persist in the host by avoiding phagocytosis (Zaragoza et al. 2010; Min et al. 2020). We suggest that the rigid Δ*sscA* conidia with accumulated glucan and chitin may impede engulfment by phagocytes and could subsequently evade the immune response to germinate into hyphae and form a biofilm, thereby leading to hypervirulence in the mice model (van de Veerdonk et al. 2017). However, further studies are required to fully comprehend the roles of SscA in the interaction between *A. fumigatus* spores and host immune cells.

Overall, our phenotypic and transcriptomic studies revealed that the spore-specific TF SscA has pleiotropic roles in the regulation of conidia formation, spore stress response and longevity, conidial wall organisation and mycotoxin production in *A. fumigatus*. The importance of SscA in conidial properties is clear but has yet to be fully investigated. Therefore, further studies are needed to identify direct targets of SscA and elucidate the detailed molecular mechanisms of SscA in *A. fumigatus* conidia.

## Supplementary Material

Supplemental Material

## Data Availability

All RNA-seq data files are available at the NCBI Sequence Read Archive (SRA) database under the accession number PRJNA983063 for WT and Δ*sscA* of *Aspergillus fumigatus* conidia RNA-seq.
