# Supplementary material for: Pleiotropic functions of SscA on the asexual spore of the human pathogenic fungus Aspergillus fumigatus
Source: Mycology. 2023 Dec 25;15(2):238–54. doi: 10.1080/21501203.2023.2294061 (PMC11132850; doi:10.1080/21501203.2023.2294061)

**Supplemental files**

**Table S1**. The SscA homologous proteins used in the phylogenetic tree.

**Table S2.** Gene Ontology (GO) term enrichment analysis of genes differentially expressed in *A. fumigatus* conidia of WT and Δ*sscA* (Biological process).

**Table S3.** Summary of differentially expressed genes involved in secondary metabolism in *A. fumigatus* conidia of WT and Δ*sscA*.

**Figure S1. Generation and verification of *sscA* deletion and complementary strains.**  (A) Colony phenotype of WT, Δ*sscA* and Cʹ *sscA* strains. At least three independent deletion mutant strains and an independent complementary strain were generated. (B) Schematic illustration of strategy used to generate Δ*sscA* or C′ *sscA* strains. Below pictures indicated PCR and qPCR verification.

**Figure S2.** **Alignment of C_2_H_2_ zinc finger domain of the SscA homologous proteins in *Aspergillus* species.** Yellow box indicated the sites of cysteine and histidine sequences in C_2_H_2_ zinc finger domain. Blue box represented different sequences of C_2_H_2_ zinc finger domain among *Aspergillus* species. The consensus amino-acid sequences were presented below (conservation rate = 100 %).

**Figure S3. The effect of SscA on germination.** (A) The microscopic images of spore germination of WT, Δ*sscA* and Cʹ *sscA* strains inoculated onto solid MMY after incubating 6 or 8 hours, respectively (bar = 50 μm). (B) The line plot showing conidial germination rate (%) of WT, *sscA*, and C' *sscA* strains.

**Figure S4. The expression patterns of α-1,3-glucan biosynthetic genes in Δ*sscA* conidia.** Heatmap plot showing mRNA expression levels of α-1,3-glucan biosynthetic genes between conidia of *A. fumigatus* wild-type and Δ*sscA*.

**Figure S5. The function of SscA in gliotoxin production in mycelia.** (A) TLC of gliotoxin from the 7-dyas grown mycelia of WT, Δ*sscA* and Cʹ *sscA* strains. (B) Relative bend intensity of gliotoxin produced in the 7-dyas grown mycelia of WT, Δ*sscA* and Cʹ *sscA* strains; error bars indicate the standard error of the mean in three biological replicates (****p* < 0.001).

**Figure S6. Transcriptomic analyses of secondary metabolites biosynthetic genes in Δ*sscA* conidia.** Heatmap diagram showing relative transcript abundance of genes involved in nidulanin-like (A), ferricrocin (B), trypacidin (C) or fumiquinozalines (D) in *sscA* deletion strain as compared with wild-type strain.

**Figure S7. Transcriptomic analyses of stress-related genes in WT and Δ*sscA* conidia.** (A-B) Heatmap plot showing mRNA expression levels of small heat-shock proteins (B) or DNA repair (A) related genes between conidia of *A. fumigatus* WT and Δ*sscA*.

**Table S1. The SscA homologous proteins used in the phylogenetic tree.**

| **Subgenus** | **Section** | **Series** | **Species** | **NCBI accession** |
| --- | --- | --- | --- | --- |
| *Aspergillus* | *Aspergillus* | *Aspergillus* | *A. glaucus* CBS 516.65 | XP 022402455.1 |
|  |  | *Rubri* | *A. ruber* CBS 135680 | XP 040641915.1 |
|  |  | *Chevalierorum* | *A. cristatus* | ODM19105.1 |
|  |  |  | *A. chevalieri* | XP 043134004.1 |
| *Circumdati* | *Candidi* | *Candidi* | *A. taichungensis* | PLN80449.1 |
|  |  |  | *A. campestris* IBT 28561 | XP 024696833.1 |
|  |  |  | *A. candidus* | XP 024674873.1 |
|  | *Circumdati* | *Circumdati* | *A. melleus* | XP 045939288.1 |
|  |  |  | *A. affinis* | XP 052952614.1 |
|  |  | *Steyniorum* | *A. steynii* IBT 23096 | XP 024708354.1 |
|  | *Flavi* | *Alliacei* | *A. alliaceus* | XP 031895158.1 |
|  |  | *Avenacei* | *A. avenaceus* | KAE8146447.1 |
|  |  | *Bertholletiarum* | *A. bertholletiae* | KAE8380017.1 |
|  |  | *Coremiiformes* | *A. coremiiformis* | KAE8349239.1 |
|  |  | *Flavi* | *A. arachidicola* | KAE8335215.1 |
|  |  |  | *A. novoparasiticus* | KAB8218074.1 |
|  |  |  | *A. sergii* | KAE8323399.1 |
|  |  |  | *A. minisclerotigenes* | KAB8273120.1 |
|  |  |  | *A. flavus* NRRL3357 | XP 041140702.1 |
|  |  |  | *A. oryzae* RIB40 | XP 001818207.1 |
|  |  |  | *A. parasiticus* | KAB8203566.1 |
|  |  |  | *A. transmontanensis* | KAE8312300.1 |
|  |  | *Kitamyces* | *A. caelatus* | XP 031932552.1 |
|  |  |  | *A. pseudocaelatus* | KAE8410350.1 |
|  |  |  | *A. pseudotamarii* | XP 031912334.1 |
|  |  |  | *A. tamarii* | KAE8162697.1 |
|  |  | *Leporum* | *A. hancockii* | KAF7592026.1 |
|  |  |  | *A. leporis* | KAB8071915.1 |
|  |  | *Nomiarum* | *A. nomiae* NRRL 13137 | XP 015402200.1 |
|  |  |  | *A. pseudonomiae* | XP 031935998.1 |
|  | *Nigri* | *Carbonari* | *A. ibericus* CBS 121593 | XP 025574167.1 |
|  |  |  | *A. sclerotiicarbonarius* CBS 121057 | PYI07478.1 |
|  |  |  | *A. carbonarius* ITEM 5010 | OOF96634.1 |
|  |  |  | *A. sclerotioniger* CBS 115572 | XP 025467673.1 |
|  |  | *Heteromorphi* | *A. heteromorphus* CBS 117.55 | XP 025404308.1 |
|  |  |  | *A. ellipticus* CBS 707.79 | PYH89454.1 |
|  |  | *Homomorphi* | *A. homomorphus* CBS 101889 | XP 025550044.1 |
|  |  | *Japonici* | *A. saccharolyticus* JOP 1030-1 | XP 025429555.1 |
|  |  |  | *A. japonicus* CBS 114.51 | XP 025524056.1 |
|  |  |  | *A. uvarum* CBS 121591 | XP 025490216.1 |
|  |  |  | *A. aculeatus* ATCC 16872 | XP 020055579.1 |
|  |  |  | *A. aculeatinus* CBS 121060 | XP 025500713.1 |
|  |  |  | *A. brunneoviolaceus* CBS 621.78 | XP 025436171.1 |
|  |  | *Nigri* | *A. costaricaensis* CBS 115574 | XP 025542856.1 |
|  |  |  | *A. tubingensis* CBS 134.48 | OJI83809.1 |
|  |  |  | *A. neoniger* CBS 115656 | XP 025484521.1 |
|  |  |  | *A. vadensis* CBS 113365 | XP 025558933.1 |
|  |  |  | *A. luchuensis* CBS 106.47 | OJZ86720.1 |
|  |  |  | *A. piperis* CBS 112811 | XP 025511196.1 |
|  |  |  | *A. eucalypticola* CBS 122712 | XP 025388012.1 |
|  |  |  | *A. niger* CBS 513.88 | XP 001397661.1 |
|  |  |  | *A. welwitschiae* | XP 026627564.1 |
|  |  |  | *A. brasiliensis* CBS 101740 | OJJ71983.1 |
|  | *Tannerorum* | *Tannerorum* | *A. tanneri* | XP 033425449.1 |
|  | *Terrei* | *Terrei* | *A. terreus* NIH2624 | XP 001213684.1 |
| *Cremei* | *Cremei* | *Wentiorum* | *A. wentii* DTO 134E9 | XP 040692776.1 |
| *Fumigati* | *Clavati* | *Clavati* | *A. clavatus* NRRL 1 | XP 001270982.1 |
|  | *Fumigati* | *Fumigati* | *A. fumigatus* Af293 | XP 754637.2 |
|  |  |  | *A. fischeri* NRRL 181 | XP 001263478.1 |
|  |  |  | *A. lentulus* | XP 033415143.1 |
|  |  |  | *A. fumigatiaffinis* | KAF4212031.1 |
|  |  |  | *A. novofumigatus* IBT 16806 | XP 024680595.1 |
|  |  | *Thermomutati* | *A. thermomutatus* | XP 026618725.1 |
|  |  | *Unilaterales* | *A. turcosus* | RHZ66550.1 |
|  |  |  | *A. hiratsukae* | KAF7121591.1 |
|  |  | *Viridinutantes* | *A. felis* | KAF7173503.1 |
|  |  |  | *A. pseudoviridinutans* | XP 043158351.1 |
|  |  |  | *A. udagawae* | GFF92317.1 |
|  |  |  | *A. viridinutans* | XP 043124607.1 |
| *Nidulantes* | *Cavernicolarum* | *Egyptiaci* | *A. egyptiacus* | KAI9373699.1 |
|  | *Nidulantes* | *Multicolores* | *A. mulundensis* | XP 026605972.1 |
|  |  | *Nidulantes* | *A. nidulans* FGSC A4 | XP 662607.1 |
|  |  | *Versicolores* | *A. puulaauensis* | XP 041554786.1 |
|  |  |  | *A. sydowii* CBS 593.65 | XP 040704807.1 |
|  |  |  | *A. versicolor* CBS 583.65 | XP 040666081.1 |
|  | *Ochraceorosei* | *Ochraceorosei* | *A. ochraceoroseus* IBT 24754 | XP 040748816.1 |
|  |  |  | *A. rambellii* | KKK26486.1 |
|  | *Usti* | *Calidousti* | *A. calidoustus* | CEL07901.1 |
|  |  |  | *A. carlsbadensis* | KAJ0424071.1 |
| *Polypaecilum* | *Polypaecilum* | *Noonimiarum* | *A. sclerotialis* | RJE20145.1 |

**Table S2. Gene Ontology (GO) term enrichment analysis of genes differentially expressed in *A. fumigatus* conidia of WT and Δ*sscA* (Biological process).**

| **GO ID** | **GO name** | **Annotated** | **Significant** | **Expected** | **classicFisher** | **Ratio** | |
| --- | --- | --- | --- | --- | --- | --- | --- |
| **UP** | | | | | | |  |
| GO:0048856 | Anatomical structure development | 409 | 78 | 54.60 | 0.000560 | 0.190709 | |
| GO:0044550 | Secondary metabolite biosynthetic process | 417 | 80 | 55.67 | 0.000400 | 0.191847 | |
| GO:0044255 | Cellular lipid metabolic process | 267 | 55 | 35.64 | 0.000560 | 0.205993 | |
| GO:0030448 | Hyphal growth | 190 | 42 | 25.36 | 0.000560 | 0.221053 | |
| GO:1901615 | Organic hydroxy compound metabolic process | 185 | 41 | 24.70 | 0.000610 | 0.221622 | |
| GO:0071554 | Cell wall organization or biogenesis | 194 | 43 | 25.90 | 0.000460 | 0.221649 | |
| GO:0048315 | Conidium formation | 130 | 30 | 17.35 | 0.001620 | 0.230769 | |
| GO:0061794 | Conidium development | 130 | 30 | 17.35 | 0.001620 | 0.230769 | |
| GO:0045229 | External encapsulating structure organization | 116 | 28 | 15.49 | 0.001110 | 0.241379 | |
| GO:0072330 | Monocarboxylic acid biosynthetic process | 102 | 25 | 13.62 | 0.001590 | 0.245098 | |
| GO:0051301 | Cell division | 100 | 25 | 13.35 | 0.001180 | 0.250000 | |
| GO:0005975 | Carbohydrate metabolic process | 409 | 103 | 54.60 | 0.000000 | 0.251834 | |
| GO:0032989 | Cellular component morphogenesis | 101 | 27 | 13.48 | 0.000240 | 0.267327 | |
| GO:0046165 | Alcohol biosynthetic process | 81 | 22 | 10.81 | 0.000700 | 0.271605 | |
| GO:0097435 | Supramolecular fiber organization | 71 | 20 | 9.48 | 0.000730 | 0.281690 | |
| **GO ID** | **GO name** | **Annotated** | **Significant** | **Expected** | **classicFisher** | **Ratio** | |
| **DOWN** | | | | | | |  |
| GO:1901361 | Organic cyclic compound catabolic process | 150 | 20 | 12.98 | 0.033800 | 0.133333 | |
| GO:0019439 | Aromatic compound catabolic process | 132 | 18 | 11.42 | 0.035100 | 0.136364 | |
| GO:0009063 | Cellular amino acid catabolic process | 53 | 9 | 4.59 | 0.036500 | 0.169811 | |
| GO:1901657 | Glycosyl compound metabolic process | 45 | 9 | 3.89 | 0.013500 | 0.200000 | |
| GO:0009116 | Nucleoside metabolic process | 40 | 9 | 3.46 | 0.006100 | 0.225000 | |
| GO:0009895 | Negative regulation of catabolic process | 17 | 4 | 1.47 | 0.053300 | 0.235294 | |
| GO:0015807 | L-amino acid transport | 16 | 4 | 1.38 | 0.043700 | 0.250000 | |
| GO:0009119 | Ribonucleoside metabolic process | 23 | 6 | 1.99 | 0.011600 | 0.260870 | |
| GO:0006490 | Oligosaccharide-lipid intermediate | 14 | 4 | 1.21 | 0.027600 | 0.285714 | |
| GO:0006857 | Oligopeptide transport | 10 | 3 | 0.87 | 0.048800 | 0.300000 | |
| GO:0006582 | Melanin metabolic process | 13 | 4 | 1.12 | 0.021100 | 0.307692 | |
| GO:0006570 | Tyrosine metabolic process | 9 | 3 | 0.78 | 0.036500 | 0.333333 | |
| GO:0042278 | Purine nucleoside metabolic process | 14 | 5 | 1.21 | 0.004900 | 0.357143 | |
| GO:0031145 | Anaphase-promoting complex-dependent | 11 | 4 | 0.95 | 0.011200 | 0.363636 | |
| GO:0006370 | 7-methylguanosine mRNA capping | 5 | 2 | 0.43 | 0.062700 | 0.400000 | |

| **Cluster number** | **Secondary metabolite** | **Number of Genes** | ***ΔsscA*** | | |
| --- | --- | --- | --- | --- | --- |
|  |  |  | **Up** | **Down** |  |
| 1 | Unknown | 5 | 2 | 0 |  |
| 2 | Nidulanin-like | 12 | 8 | 2 |  |
| 3 | Ferricrocin | 2 | 2 | 0 |  |
| 4 | Fusarielin-like | 6 | 1 | 0 |  |
| 5 | Unknown | 16 | 5 | 2 |  |
| 6 | Unknown | 16 | 6 | 3 |  |
| 7 | DHN melanin | 6 | 0 | 2 |  |
| 8 | Fumigaclavine | 11 | 1 | 1 |  |
| 9 | Unknown | 9 | 3 | 0 |  |
| 10 | Unknown | 27 | 8 | 3 |  |
| 11 | Fusarine C | 6 | 1 | 0 |  |
| 12 | Unknown | 12 | 5 | 3 |  |
| 13 | Hexadehydroastechrome | 8 | 4 | 0 |  |
| 14 | Unknown | 14 | 2 | 3 |  |
| 15 | Unknown | 9 | 7 | 0 |  |
| 16 | Unknown | 6 | 2 | 1 |  |
| 17 | Endocrocin | 4 | 1 | 0 |  |
| 18 | Trypacidin | 13 | 8 | 0 |  |
| 19 | Helvolic acid | 9 | 1 | 1 |  |
| 20 | Unknown | 11 | 3 | 0 |  |
| 21 | Unknown | 10 | 2 | 2 |  |
| 22 | Fumisoquin | 7 | 3 | 0 |  |
| 23 | Unknown | 5 | 3 | 2 |  |
| 24 | Unknown | 5 | 2 | 1 |  |
| 25 | Gliotoxin | 12 | 3 | 2 |  |
| 26 | Fumiquinozalines | 5 | 4 | 1 |  |
| 27 | Pyripyropene A | 9 | 1 | 2 |  |
| 28 | Neosartoricin | 6 | 2 | 1 |  |
| 29 | Fumitremorgin | 9 | 1 | 2 |  |
| 30 | Fumagillin and pseurotin | 21 | 5 | 2 |  |
| 31 | Unknown | 2 | 1 | 0 |  |
| 32 | Unknown | 12 | 4 | 2 |  |
| 33 | Unknown | 5 | 0 | 1 |  |

**Table S3. Summary of differentially expressed genes involved in secondary metabolism in *A. fumgiatus* conidia of WT and Δ*sscA*.**

* Orange boxes indicate secondary metabolite, with a DEG ratio > 60 % per gene cluster.


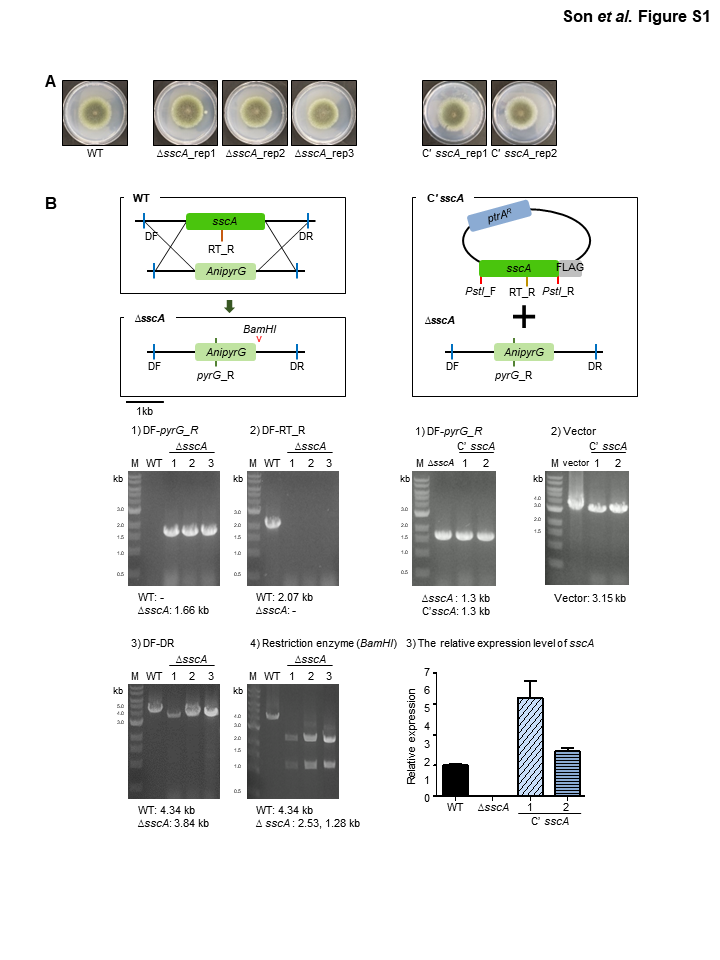


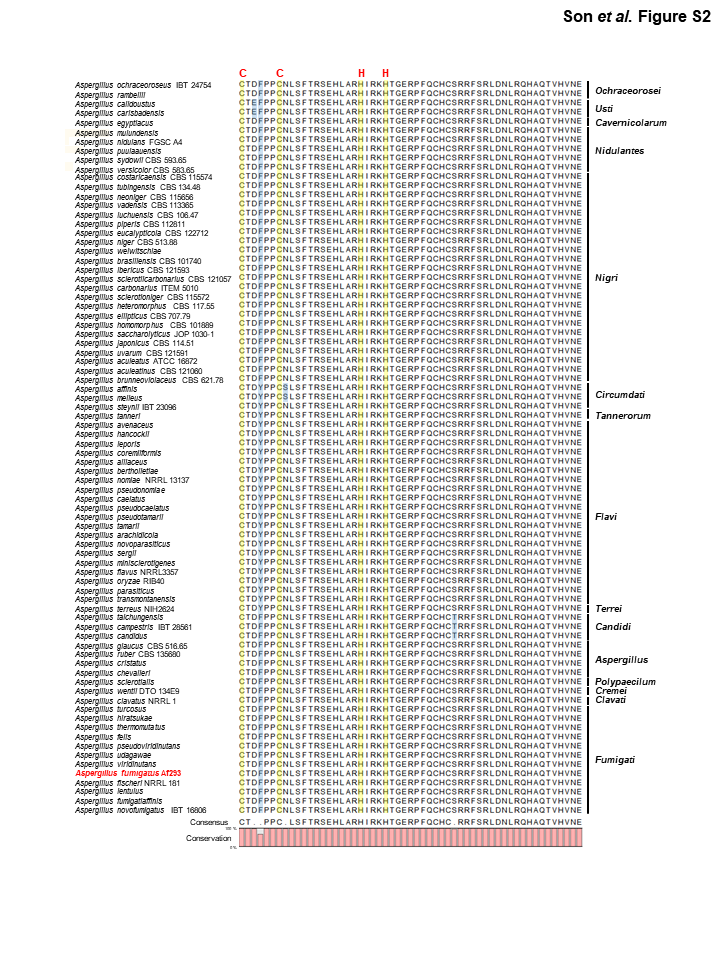

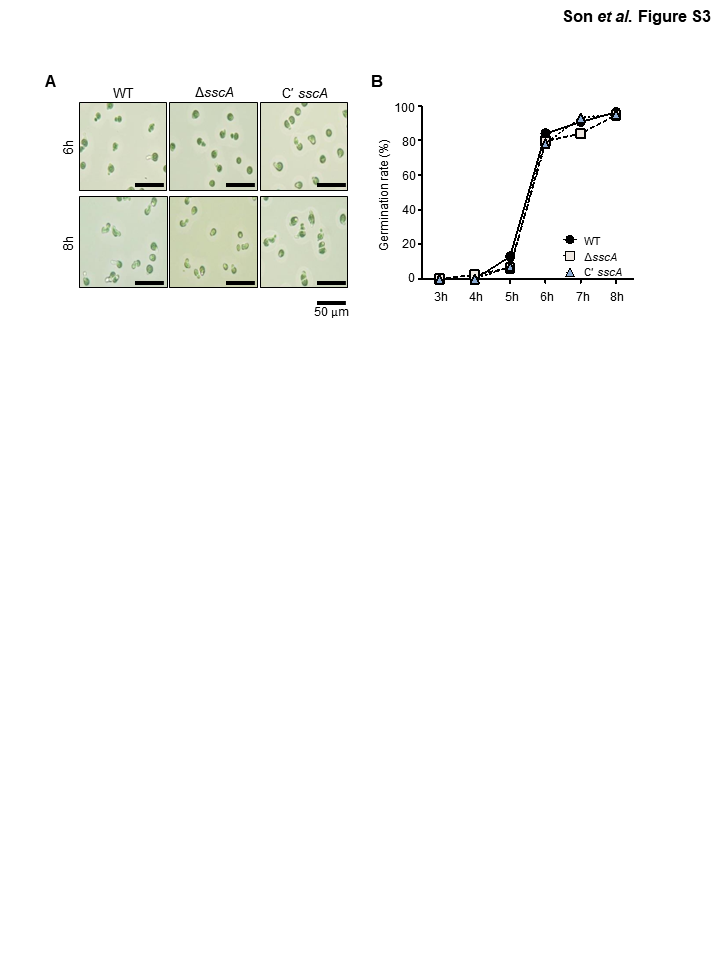

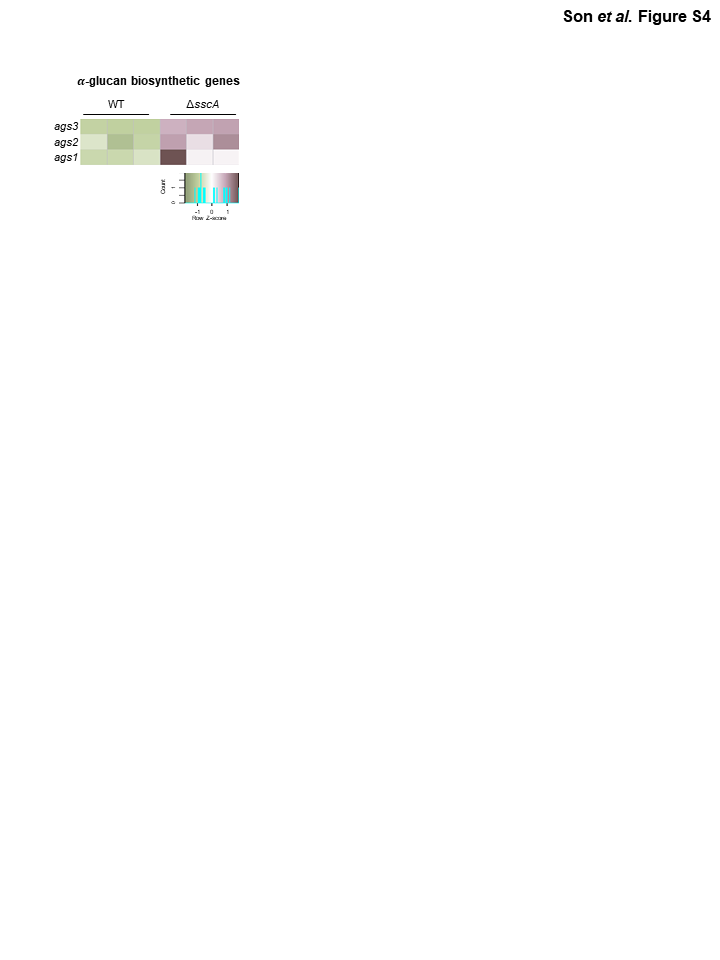

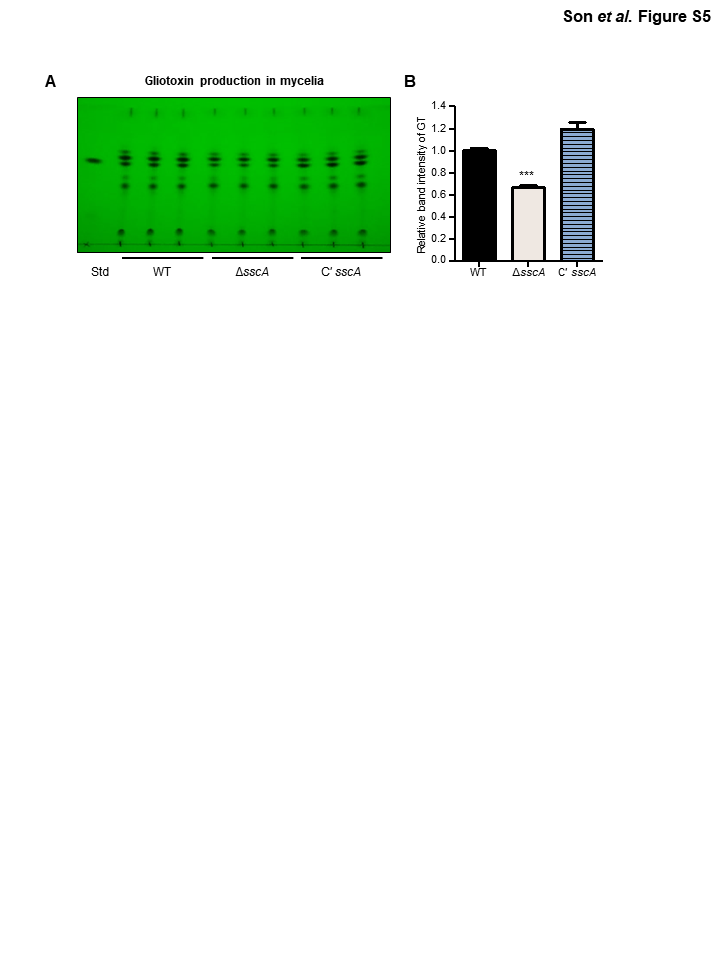

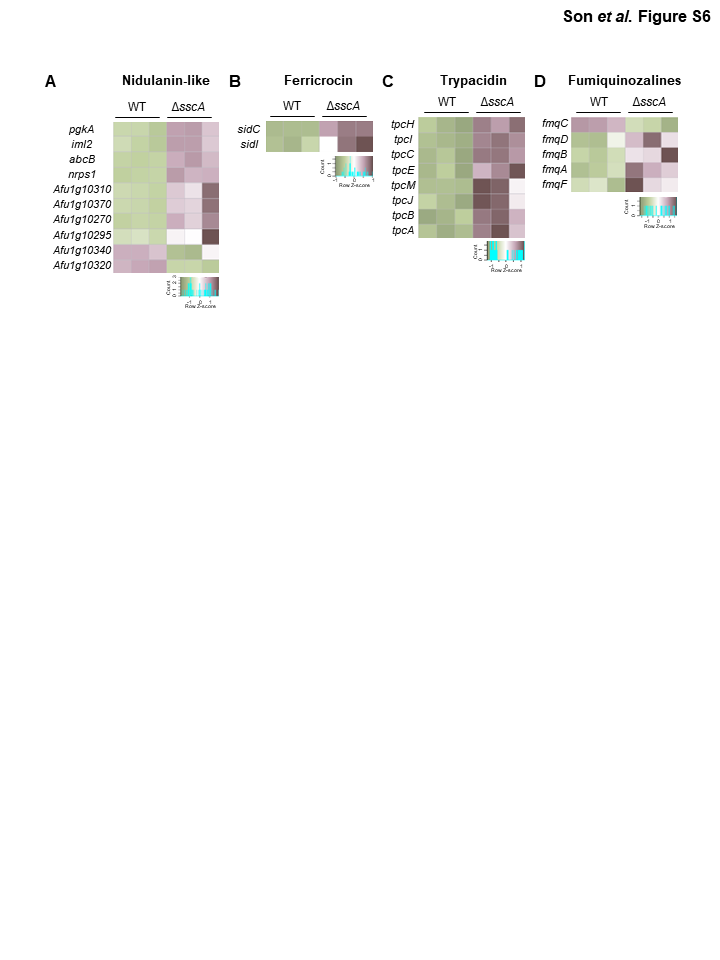

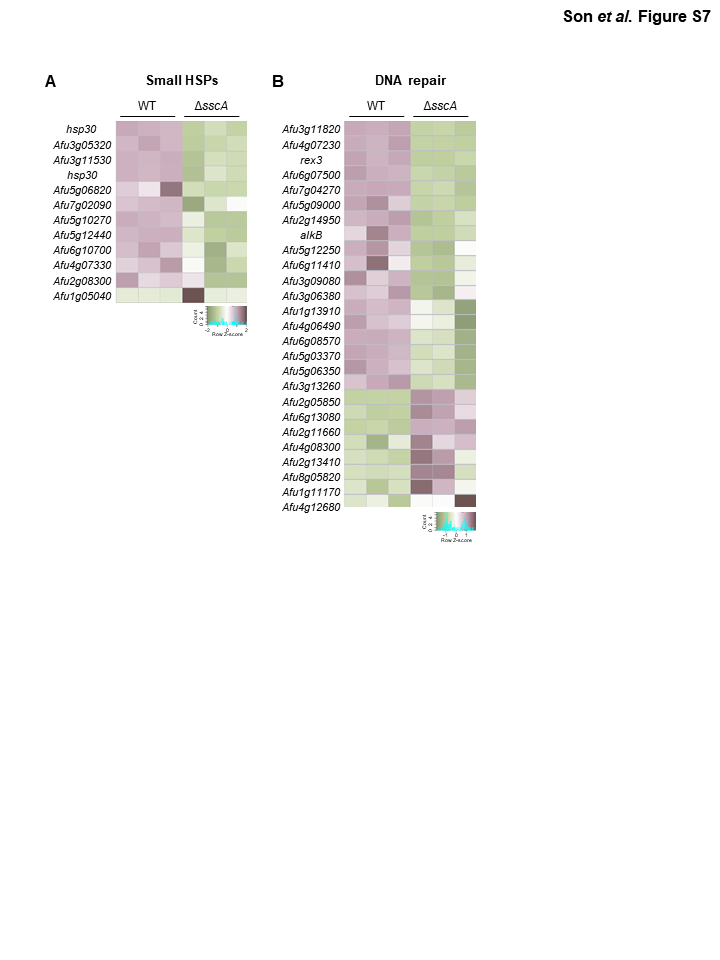

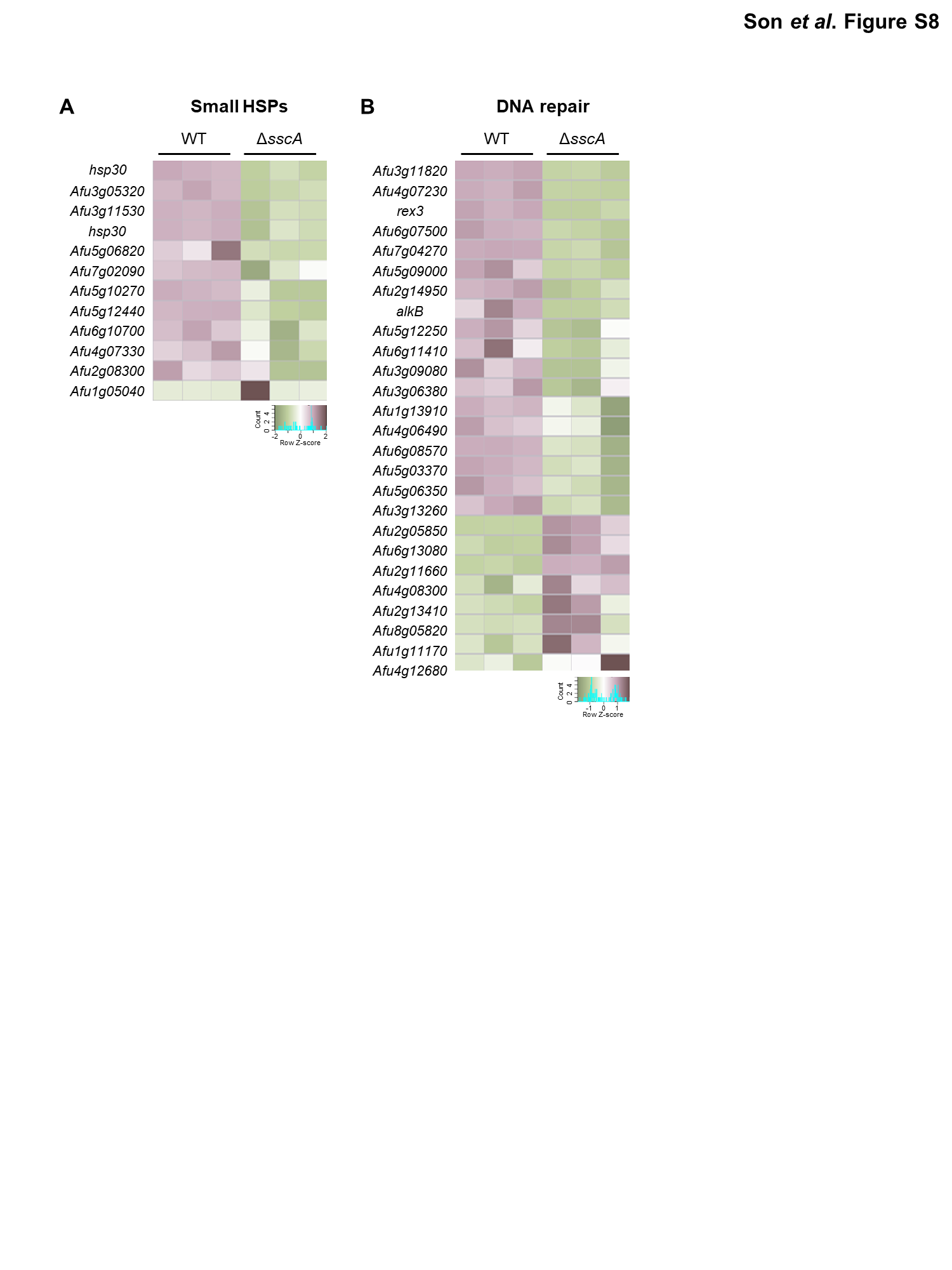

Supplement: Supplemental Material [file TMYC_A_2294061_SM5502.docx]
